# A Practical Treatise on Diseases of the Skin

**Published:** 1897-06

**Authors:** 


A PRACTICAL TREATISE ON DISEASES OF THE SKIN. For the use of Students and Practitioners. By J. NEVINS HYDE, A. M., M. D., Professor of Dermatology and Venereal Diseases in Rush Medical College, Chicago, and FRANK H. MONTGOMERY, M. D., Lecturer on Dermatology and Venereal Diseases, Rush Medical College, Chicago. New (fourth) edition. In one octavo volume of 815 pages, with 110 engravings and twelve full-page plates, four of which are colored. Philadelphia and New York: Lea Brothers & Co., Publishers. 1897.

The demand for another edition of Dr. Hyde's work on skin diseases within so short a period is a practical endorsement and evidence of value accorded to but few books. So much has been said in detail concerning it upon previous occasions that it remains only to refer to the fact that the book has been gone over from beginning to end, eliminating here and adding there, so that as the work stands it has all that is recent and that has been found practical and established. Coincidently it has been judiciously pruned of unnecessary or obsolete matter.

It is a treatise of exceptional merit, being characterised by conscientious care and scientific accuracy, and may well serve as a model for other writers on similar subjects. The author has, in his best manner, correlated the various treatments for skin affections with the reason and indications peculiar to each. It compares favorably with any work published and is a credit to the American profession. From the standpoint of the publisher is eminently satisfactory ; especially creditable are the illustrations, and we would cordially commend it to our readers. E. W.

